# SARS-CoV-2 and the host-immune response

**DOI:** 10.3389/fimmu.2023.1195871

**Published:** 2023-06-19

**Authors:** David P. Maison, Youping Deng, Mariana Gerschenson

**Affiliations:** ^1^ Department of Cell and Molecular Biology, John A. Burns School of Medicine, University of Hawaii at Manoa, Honolulu, HI, United States; ^2^ Department of Quantitative Health Sciences, John A. Burns School of Medicine, University of Hawaii at Manoa, Honolulu, HI, United States

**Keywords:** COVID-19, immune response, innate immunity, adaptive immunity, viral immune evasion

## Abstract

The SARS-CoV-2 pandemic and the COVID-19 disease have affected everyone globally, leading to one of recorded history’s most significant research surges. As our knowledge evolves, our approaches to the virus and treatments must also evolve. The evaluation of future research approaches to SARS-CoV-2 will necessitate reviewing the host immune response and viral antagonism of that response. This review provides an overview of the current knowledge on SARS-CoV-2 by summarizing the virus and human response. The focuses are on the viral genome, replication cycle, host immune activation, response, signaling, and antagonism. To effectively fight the pandemic, efforts must focus on the current state of research to help develop treatments and prepare for future outbreaks.

## Introduction

SARS-CoV-2 emerged in November 2019 in Wuhan, China, from multiple zoonotic cross-species transmission events in humans. The subsequent COVID-19 pandemic - declared by the WHO on March 11, 2020 - is ongoing, and has seen substantial viral evolution and new variants (1–9). Between November 2019 and November 2022, over 632 million cases of COVID-19 were confirmed globally, with more than 6.6 million confirmed deaths and models predicting nearly 20 million deaths (10, 11).

Our understanding of the host immune response to SARS-CoV-2 is constantly growing, as are the discoveries related to immune modulatory effects related to the virus and viral proteins. This review will cover the SARS-CoV-2 genome, virion structure, viral entry and replication, the host immune response, and SARS-CoV-2 immune antagonism. Understanding the host immune response and viral immune antagonism is crucial - as the current state of research - as this knowledge can guide novel treatment strategies and inform public health measures.

## SARS-CoV-2 genome and virion structure

**Figure 1:**
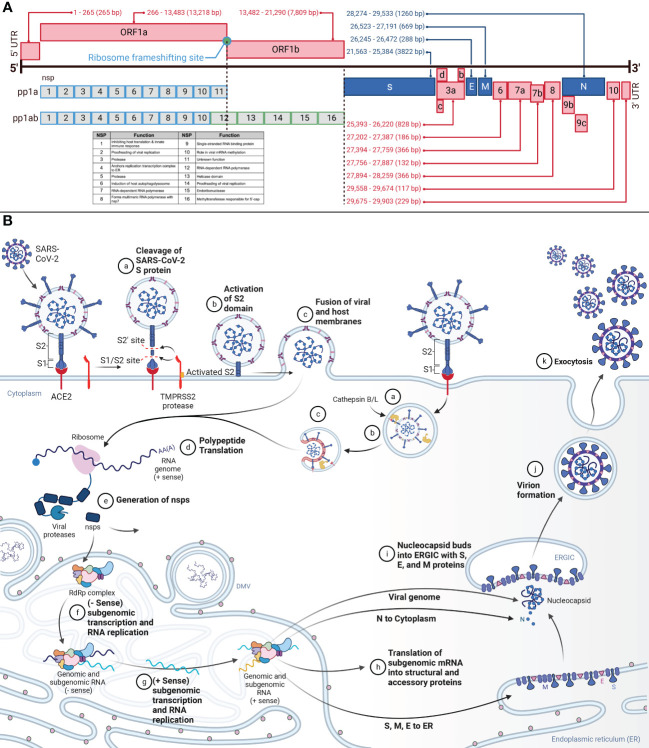
**(A)** Genomic Organization of SARS-CoV-2 and SARS-CoV-2 Viral Entry and Replication Cycle. This figure demonstrates the genome of SARS-CoV-2, a positive-sense RNA virus, and the proteins produced from each genome segment. The 29.903 kilobase genome was first identified in Wuhan, China, in December 2019. From 5’ to 3’, the genome is organized as 5’-cap structure, 5’ UTR, ORF1ab, spike (S), ORF3a-d, envelope (E), membrane (M), ORF6, ORF7a-b, ORF8, ORF9b-c, nucleocapsid (N), ORF10, 3’ UTR, and 3’-poly-A tail. Nsps 1-16 are translated from ORF1ab. Structural and accessory proteins are translated from their corresponding segment. Nucleotide annotations acquired from GenBank Accession NC_045512. **(B)** This figure represents the overview of SARS-CoV-2 viral entry and the replication cycle. aSARS-CoV-2 entry begins after binding the S protein S1 domain to the ACE2. The S1/S2 site on the S protein will then be cleaved by TMPRSS2, resulting in the removal of S1. bFollowing, the S2’ site is cleaved by TMPRSS2, furin, and cathepsins, priming the S protein by allowing S2 to form a pre-hairpin in the cellular membrane. cThe pre-hairpin forms a six-hairpin bundle that pulls the viral membrane and cellular membranes together, fusing them and allowing the viral RNA to release into the cytosol. dUpon release into the cytosol, translation of the viral RNA will begin to be translated. eThe non-structural proteins (nsps) polyproteins pp1a and pp1ab will be translated from ORF1ab. Autoproteolysis and post-translational processing will form the replication transcription complex (RTC) and the RNA-dependent-RNA-polymerase (RdRp) within the endoplasmic reticulum (ER). fWithin the ER, in specialized double-membrane vesicles (DMV), the RTC will synthesize the complementary negative-strand genomes and negative-strand subgenomic RNAs. gThe negative strands will serve as the template for progeny genomes and subgenomic RNAs. hThe subgenomic RNAs are translated as structural and accessory proteins. The structural proteins S, E, and M are translated into the ER membrane and the N into the cytoplasm to encapsulate the viral genome. iThe N-coated viral genome will bud into the ERGIC complex containing S, E, and M proteins. jThe virion will be budded into a vesicle bound to the cellular membrane. kThe vesicle will fuse with the cellular membrane, and exocytosis will release the fully-formed virus into extracellular space.

SARS-CoV-2 is an enveloped, positive-sense RNA virus of the family *coronaviridae (* 12 *).* Within the *coronaviridae* family, SARS-CoV-2 is part of the betacoronavirus genera, also known as genus sarbecoviruses (13). The 29.903 kilobase SARS-CoV-2 genome was published in January 2020 (3, 5, 14). The ancestral lineage A and B genomes are ~80% similar to SARS-CoV and 96.2% similar to the Bat RaTG13 virus (4, 14, 15). From 5’ to 3’, the genome is organized as 5’-cap structure, 5’ UTR, ORF1ab, S, ORF3a-d, E, M, ORF6, ORF7a-b, ORF8, ORF9b-c, N, ORF10, and 3’ UTR (**Figure 1A**) (13, 16–19). The genome contains coding for four structural proteins, 11 accessory proteins, and 16 nsps (12). The four structural genes are S, E, M, and N. The structural proteins and the genome form an enveloped virion able to infect cells (15). The 11 accessory genes and proteins are ORF3a-d, ORF6, ORF7a and b, ORF8, ORF9b and c, and ORF10, and serve various functions from host interaction to immune modulation (18–20). The 16 nsps are identified numerically as nsp1-16 (12). All the nsps are synthesized from ORF1a and ORF1ab (12, 15–17). The change from ORF1a to ORF1ab is facilitated by the ribosomal frameshift of a stop codon at the end of ORF1a (16, 17).

The structural proteins form the virus’s envelope and hold the RNA genome. The N protein coats the genome, whereas S, M, and E are embedded in the lipid bilayer membrane (16). The N protein is composed of positively charged amino acids and binds the negatively charged backbone of genomic RNA (12). The S protein is a 1273 amino acid protein divided into S1 and S2 portions (21), and domains (17, 22). S1 contains the NTD and the RBD (22). Juxtaposed is the S1/S2 furin cleavage site. S2 includes the S2’ cleavage site, FP, HR1, CH, HR2, TM, and the cytoplasmic tail (22, 23). Spike proteins complex into trimers that protrude from the viral envelope surface. On the viral envelope is an average of 15-30 of these S protein trimers (12, 24). Cellular entry by SARS-CoV-2 relies on S protein binding to ACE2 and S protein priming by TMPRSS2 (25). Cathepsin B and L can also serve the same function as TMPRSS2 but within the endosome (15, 21, 26). The E protein, a single-span transmembrane protein, facilitates viral assembly and release, and is an important component in pathogenesis (27). In addition to being transmembrane, the E protein contains channel activity, forming pores and allowing ion transport (28). The M protein is the most abundant structural protein, and spans the membrane three times and interacts with both the E and the S proteins to facilitate the structure of the lipid envelope (12, 27).

## Viral entry and replication cycle

The replication cycle (**Figure 1B**) begins after SARS-CoV-2 binding to ACE2 via the S1 subunit of the S protein (16, 25). The spike protein S1 ectodomain changes conformation from open to closed, and in the open conformation, the RBD interacts with ACE2 in humans (12, 14, 29). The S2 subunit of the S protein then facilitates membrane fusion (16). Two cleavage sites in the S protein are responsible for the pre-fusion to post-fusion conformational change (30, 31). The S1/S2 site, consisting of the polybasic furin motif, separates the S1 and S2 domains (4, 26, 32, 33). The S2’ cleavage site drives the fusion of the virus with the cell membrane (16, 34). The S1/S2 site is cleaved by TMPRSS2 and results in the removal of the S1 subunit (16, 35). The S2’ site is cleaved by TMPRSS2, furin, and cathepsins, inducing an irreversible conformational change in the S protein (16, 36). Removing the S1 subunit and activating S2 allows S2 to form a pre-hairpin that embeds into the target cellular membrane. The pre-hairpin folds back and creates a six-hairpin bundle that pulls the viral and cellular membranes together.

The genomic vRNA will release into the cytosol and uncoat (21). Immediately, the polyproteins pp1a and pp1ab will be translated from ORF1ab (21). These proteins are required for replication and viral survival. The majority of the replication machinery is in the ORF1b portion of the genome and are produced with the ORF1a to ORF1ab -1 frameshift, which occurs at a ~20-50% rate (16). These polyproteins are co- and post-translationally processed into the RTC by nsp3 (PLPro) and nsp5 (3CLpro) (13, 16, 21, 37). The RTC is responsible for replication, transcription, RNA processing, and formation of within the ER to serve as replication factories (15, 16). The viral RTC will replicate the viral genome and subgenomic mRNA for the assembly of new viruses (21).

The RTC will synthesize negative-strand full-length genomes from the positive-strand RNA genome for the subsequent generation of positive-strand progeny genomes (16). Additionally, the RTC will generate subgenomic mRNA via discontinuous transcription utilizing transcription regulatory sequences located at the 5’ end of each ORF (16). The negative-strand subgenomic mRNAs then facilitate the generation of positive-strand subgenomic mRNA. These positive-strand subgenomic mRNA then serve as translation templates for viral protein production. Replication occurs in specialized ER structures known as DMV (16, 38). Structural proteins are processed through the endoplasmic reticulum to the Golgi apparatus. Here the S, E, and M structural proteins are retained at the budding ERGIC, where interaction with N-encapsidated genomic vRNA results in the formation of secretory vesicles (21). The M protein incorporates viral components into the virions. The N protein interacts with both the genome and the E protein to enable packaging into the virion. The E protein participates in viral assembly by functioning as an ion channel and participating in membrane curvature (16). Virions are released from the cell via exocytosis.

## Host immune response

### Innate response

**Figure 2:**
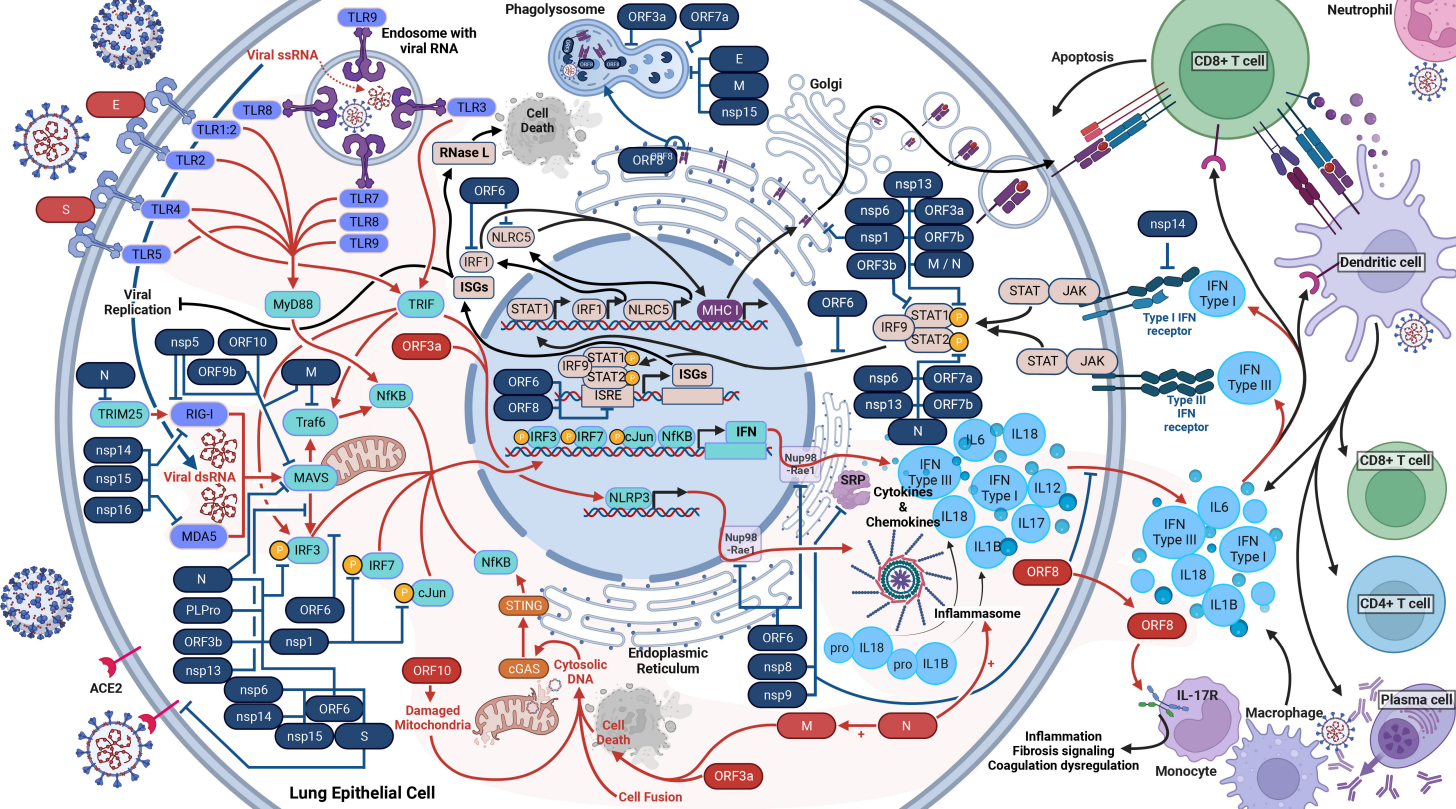
Host Immune Response to SARS-CoV-2 Infection. SARS-CoV-2 activates and antagonizes several arms of the immune response to infection. In total, SARS-CoV-2 will activate and proliferate an immune response (red arrows) through cellular damage (dysfunctional mitochondria), single-stranded RNA (ssRNA), double-stranded RNA (dsRNA), cytosolic DNA, open-reading frame 8 (ORF8), the envelope (E) protein, the spike (S) glycoprotein, and the nucleocapsid (N) protein (shown in red). These parts of the virus initiate the response to induce the production of type I interferons (IFN), type III IFN, and inflammasomes (red lines). These cytokine responses will act in autocrine and paracrine manners to activate IFN receptors (IFNAR, IFNLR) to induce interferon-stimulated genes (ISGs), interferon regulatory factor 1 (IRF1), and NOD-like receptor family CARD domain-containing five (NLRC5) (black lines). IRF1 and NLRC5 induce major histocompatibility complex (MHC) class I expression. ISGs will inhibit viral replication as well as activate RNase L, which will cleave all cellular and viral ssRNA and induce cell death (black lines). The cytokine responses will also recruit dendritic cells, CD8+ killer T cells, and initiate the adaptive immune response. The adaptive immune response to SARS-CoV-2 includes CD8+ T cells, CD4+ T cells, and B cells (plasma cells) to produce antibodies. At least 26 SARS-CoV-2 proteins (shown in blue with blue lines) antagonize some part of the immune response.

Upon infection with SARS-CoV-2, a cell and host will begin a robust innate immune response (**Figure 2**). PRRs such as RIG-I, MDA-5, and TLRs will recognize SARS-CoV-2 within the infected cell (28, 39–42). Beginning with TLR2, it senses the SARS-CoV-2 envelope protein to initiate an immune response before viral entry and replication, causing the release of TNF-α and IFN-γ (28). TLR2 also activates the assembly of the NLRP3 inflammasome (28). TLR1 is predicted to form a heterodimer with TLR2 and has significantly higher RNA counts in severe and critical COVID-19 (28). Shifting our focus to TLR3, this receptor interacts with viral PAMPs and dsRNA (43). The TLR3 response induces both the IRF3 and NF-κB response via the TRIF-dependent pathway and increases NLRP3 expression, enabling recognition of SARS-CoV-2 and formation of an inflammasome alongside other proteins. The inflammasome results in the maturation and release of IL-1β and IL-18, triggering pyroptosis (40, 41, 44, 45). In the case of TLR4, its response is to DAMPs and PAMPs resulting from SARS-CoV-2 infection and upregulates IL-6 production via NF-κB and MAPK (44, 46, 47). TLR1, TLR4, and TLR5 have been proposed - and *in-silico* predicted - to respond to the SARS-CoV-2 spike glycoprotein (44, 45, 47), which activates the MyD88 and TRIF innate immunity signaling pathways (48). Turning to TLR7, TLR8, and TLR9, these endosomal receptors play distinct roles. TLR7 and TLR8 sense ssRNA and causes a release of IL-6, TNFα, and type I and III IFNs (42, 45). TLR9 detects viral RNA and DNA with unmethylated CpG, and mtDNA released due to SARS-CoV-2-induced cellular damage. TLR9 causes the release of cytokines, including IL-1β, IL-6, IL-10, IL-17, TNFα, and type I IFN (45).

As the immune response develops, RIG-I and MDA5, upon activation by dsRNA, interact with MAVS, which initiates the phosphorylation of IRF3 (38, 49, 50). In parallel, TLRs activate MyD88, which initiates NF-κB (16, 41). These activated responses stimulate the production of many cytokines, including types I (IFNα and IFNβ) and III IFN (IFNλ), via several pathways (39–42). As such, the IFN antiviral pathway is one of the most important innate mechanisms against viral infections (38, 51). Notably, the suppressed IFN response is a major determinant of COVID-19 clinical severity (52). Meanwhile, the host cell also produces cytokines and chemokines such as IL-6, IP-10, and TNF, resulting in the cytokine storm (28, 39, 40, 53).

In the next stage of the immune response, in autocrine and paracrine manners, type I IFNs bind to IFNAR, and type III IFNs bind to IFNLR. The binding of IFNs to IFNAR and IFNLR activates the JAK/STAT pathway, inducing the expression of MHC class I and ISG via the ISRE (16, 41, 42). ISGs repress viral replication and activate RNase L, which degrades both viral and host ssRNA, leading to cell death without allowing the virus to spread (38).

Moreover, SARS-CoV-2 infection also leads to activation of cGAS-STING signaling via cell death, cell fusion, mitochondrial stress, and DNA damage, which mediates recognition of the genome DNA from fused cells and the activation of NF-κB and subsequent upregulation of TNF and IL-6 (39, 54). cGAS-STING also stimulates the IRF3 signaling pathway (39). Lastly for the innate response, proteasomes will degrade viral proteins and present them on MHC class I proteins to facilitate cytotoxic T-cell destruction of the infected cell. The dysregulation caused by the many avenues of cytokine release ultimately damages tissues and organs (28). Inflammatory cells are also activated and migrate to the lungs, producing a compounded cytokine response to add to the cytokine storm (40).

### Adaptive response

The pro-inflammatory cytokine response recruits immune cells and begins the adaptive response (39). There are several essential facets of the adaptive immune response against SARS-CoV-2, which include plasma cells, CD4+ T helper cells, and CD8+ Killer T cells (55, 56). This section will discuss the adaptive response specific to SARS-CoV-2 infection. All three of these arms (antibodies, CD4+, and CD8+) work together to combat infection with SARS-CoV-2, and follow the recognition of the virus by dendritic cells, and migration of those activated dendritic cells to lymph nodes (57). nAbs are the most common route to immunity against SARS-CoV-2 progression to COVID-19 and are therefore essential, and a focus for vaccines elicited responses (56, 58, 59). nAbs bind the virus to prevent the virus from entering cells. Long-term protection from antibodies is facilitated by memory B cells. Memory B cells, both circulating and in bone marrow, are detectable up to 6 months after SARS-CoV-2 mRNA vaccination and a year following SARS-CoV-2 infection (24, 60). B cells differentiate into plasma cells, which produce nAbs (IgG, IgA, and IgM) within severals days after infection or vaccination (24). This differentiation can occur either in the extrafollicular region during the EF phase or in the germinal centers during the GC phase. Following the EF phase, the B cells undergo somatic hypermutation and selection in germinal centers during the GC phase and compartmentalize in the bone marrow (24). However, a unique finding in acute COVID-19 is the lack of germinal centers in lymph nodes. Germinal centers are essential for developing and differentiating memory B cells and plasma cells with high-affinity antibodies (61). During COVID-19 disease, there is an absence of Bcl-6 transcription factor expressing B cells, a critical transcription factor for B cell development in germinal centers (62). This absence correlates with the lack of germinal centers in acute COVID-19, which results in “disease-related” extrafollicular B cells. The “disease-related” B cells result from class-switching and not selection in germinal centers. This class of B cells does not impart long-lasting protection. Thus, the development of B cells underscores the importance of vaccination in generating a high-affinity nAb response and protecting against COVID-19 (62).

CD4+ T helper cells are vital to the antibody responses through interactions with B cells, and are part of almost all infections with SARS-CoV-2 (55, 56). CD4+ T cell responses are more prominent than CD8+ in SARS-CoV-2 infection, are strongly associated with lessened disease severity, and have been demonstrated against 21 SARS-CoV-2 proteins; prominently S, M, N, nsp3, nsp4, nsp12, ORF3a, ORF7a, and ORF8 (55, 63–65). Memory CD4+ T cells persist and can generate a response on secondary challenge, circulate by 30 days post-symptom onset and have a half-life of 94 days (64). There are several cell functions for CD4+ seen in COVID-19 (55). The specialized subset of CD4+ T cells known as T_FH_ are important for helping generate nAbs and help B cells. T_FH_ are detectable and durable for more than six months post-symptom onset (24, 64). However, Bcl-6^+^ GC-T_FH_, a requirement for inducing germinal centers, are decreased in COVID-19. Instead, there is a robust T_H1_ response in COVID-19 (62). The T_H1_ response is involved in cytokine secretion (IFNγ) and innate cell recruitment (55). Even so, the host humoral immune response evolves continuously via affinity maturation with CD4+ T cells in germinal centers after viral resolution (66). Additionally, slower decay rates of antibodies and a higher-affinity antibody response correlate with a higher frequency of CD4+ T cells (66). T_RM_ are those memory T cells that are non-recirculating and persist in tissues, and serve to limit re-infection (67). T_RM_ in the respiratory tract exists for ten months post-infection (67). IL-22 secreting CD4+ T-cells play a role in mucosal wound healing (55).

CD8+ T cells play an essential role in many viral infections and are responsible for destroying infected cells (56). CD8+ T cell responses have been recognized against SARS-CoV-2 S protein, M protein, N protein, nsp6, and ORF3a (63–65). Memory CD8+ T cells circulate by 20-50 days post-symptom onset and have a half-life of 225 days. The preponderance of these circulating CD8+ T cells are T_EMRA_, with lesser amounts of T_EM_ and T_CM_. T_EMRA_ plays a role in protection against severe disease, as shown in other viral infections (64).

Upon infection with SARS-CoV-2, those able to generate immune responses using B cells, CD4+ cells, and CD8+ cells are able to limit disease severity (56). Further, developing a robust response with these three arms was inversely correlated with age (56). The broad spectrum of all three responses defines the adaptive immune response to SARS-CoV-2 infection and lessens the disease severity (56). An important consideration and implication for vaccine design is the inclusion of M and N to better mimic natural SARS-CoV-2 CD4+ T cell and CD8+ T cell responses (65). In vaccinated individuals, regardless of vaccine platform, memory CD4+ T cells and memory CD8+ T cells are preserved and not impacted by evolving variants. Whereas memory B cells recognition of spike proteins, and antibody reactivity, are significantly reduced against variants in both vaccinated and naturally infected persons (68, 69).

## Viral modulation of the innate response

We are now delving into the role of SARS-CoV-2 modulation or antagonism of the immune system, which SARS-CoV-2 is very effective at (55). Firstly, we will cover the structural proteins. Inside the cell, the Spike protein will antagonize the immune response by interacting with IRF3 (70). In the case of the M protein, it antagonizes innate immunity by inhibiting the TRAF complex, which is involved in the promoter activation of NF-κB and subsequent IFN transcription (16, 71). Additionally, the M protein interacts with MAVS, impairing the IFN downstream response, and further, blocks phosphorylation of STAT1, an element responsible for inducing ISG (72, 73). Lastly, the M protein triggers cell apoptosis with the N protein as a cofactor (74). Switching to the N protein, it targets the initiation of RIG-1 pathway via blocking TRIM25, and additionally prevents phosphorylation of STAT1, STAT2, and IRF3, blocking the entry of all three into the nucleus and inhibiting the IFN and ISG responses (16, 51). The N protein is also shown to prevent the aggregation of MAVS, as well as promote the activation and assembly of the inflammasome (75, 76).

Now moving on to the non-structural proteins. Nsp1 blocks the phosphorylation of IRF3, IRF7, STAT1, and cJun. Nsp1 additionally directly inhibits the IFN and NF-κB promoters and interacts with host 40S ribosomal subunits via 18S rRNA to inhibit host protein translation. Further, nsp1 degrades transcripts lacking a 5’ viral leader sequence (16, 38, 52, 77–80). Regarding nsp3, it blocks cytokine production (81). Nsp3, like N protein, also block the phosphorylation of IRF3, preventing nuclear translocation, and antagonize type I IFN activity (16, 81, 82). Transitioning to nsp5, it proteolytically cleaves RIG-I and induces the degradation of MAVS, thus preventing detection of viral dsRNA and inhibiting the IFN pathway (83). In the case of nsp8 and nsp9, they bind to the SRP and disrupt protein trafficking, suppressing the type I IFN response (52). As for nsp10, it impairs the activity of IRF3 and NF-κB binding sites (81). Exploring nsp6 and nsp13, they bind to an intermediary between MAVS and IRF3 signaling; thus limiting IRF3 activation (63, 73, 77, 84). Nsp6 and nsp13 also inhibit phosphorylation of STAT1 and STAT2 (73). Additionally, nsp13 limits nuclear translocation of NF-κB (77). Nsp13, nsp14, nsp15, and ORF6 prevent nuclear translocation of IRF3 (85). Turning to nsp14, nsp15, and nsp16, they modify the viral RNA and prevent recognition by RIG-1 and MDA-5 (38). Nsp14 additionally targets the IFNAR receptor for lysosomal degradation (81). Lastly, nsp16 additionally binds to the spliceosome and interrupts mRNA splicing, further suppressing the IFN response (52).

Now we move on to the ORFs. ORF3a blocks the phosphorylation of STAT1, activates the NLRP3 inflammasome, prevents phagosome and lysosome fusion, and induces cell death via the extrinsic apoptosis pathway (18, 20, 73, 81). ORF3b antagonizes type I IFN activity (86). Regarding ORF6, it blocks the translocation of IRF3 and the STAT1 complex into the nucleus and inhibits the MHC class I pathway (16, 73, 87). ORF6 also binds to the interferon-inducible nuclear export complex of Nup98 and Rae1, preventing the nuclear release of mRNA (63, 84). Moreover, ORF6, ORF3b and ORF8 inhibit the ISRE to type I IFN production (38, 88). Focusing on ORF7a, it inhibits the translocation of STAT2 to the nucleus, reduces phagolysosome acidity, and binds to monocytes, decreasing their ability to present antigens (18, 73, 81, 89). In the case of ORF7b, it suppresses STAT1 and STAT2 phosphorylation (73). Turning to ORF8, it directly interacts with MHC class I proteins on the ER membrane and facilitates their degradation via autophagosome degradation (90). Additionally, ORF8 is a secreted protein that mimics IL17A and interacts with IL17 receptors on monocytes. This interaction of monocytes upregulates gene expression in fibrosis signaling, coagulation dysregulation, and inflammation (91). Examining ORF9b, it interacts with Tom70, thereby interfering with MAVS and type I IFN expression (84, 92). As for ORF9c, it upregulates IL-6 signaling while impairing IFN signaling (93). Lastly, ORF10 induces mitophagy and thereby causes the degradation of MAVS (94).

## Conclusions

The rapid emergence and spread of SARS-CoV-2 worldwide has vastly changed research over the past years. This dynamic has included more than 10,000 new SARS-CoV-2 articles released per month (95). This review aims to summarize some of the most relevant and referenced immune response resources into a comprehensive summary of the current state of the host immune response to SARS-CoV-2. This amalgamation of information represents the background and current state of understanding in an ever-evolving and dynamic field. Understanding the virus-host immune interactions can provide valuable insights for developing targeted therapeutics, such as antivirals and immunomodulatory drugs, which can mitigate the severity of the infection and limit viral spread. Furthermore, this knowledge can inform pandemic preparedness by helping to identify potential therapeutic targets and designing effective pre-emptive interventions.

The topics covered herein require constant reevaluation as more evidence is published worldwide. Such reflection will allow for refining the selection of drug targets and elucidating and understanding the molecular basis of pathogenesis. Understanding the molecular pathway of SARS-CoV-2 viral inhibition of IFN production and signaling for drug targets and pathogenesis can point to other prospective treatments, such as IFN mimicry, small molecules, solubilizable ACE2 in place of monoclonal antibodies (96), prophylactic RIG-I agonists (97), or nanobody-fusions targeting specific ORFs. There are many currently available and approved IFN receptor agonists that clinical trials could further explore (ropeginterferon alfa-2b, peginterferon alfa-2b, etc). These points of continuous assessment allow for the most effective management of SARS-CoV-2, COVID-19, and future pandemics.

## Author contributions

DM prepared the initial manuscript. DM, YD, and MG collectively produced the final version. All authors contributed to the article and approved the submitted version.

## Glossary

**Table:**

3CLpro	chymotrypsin-like protease
ACE2	the angiotensin-converting enzyme 2
Bcl-6	B cell lymphoma 6
CD4	cluster of differentiation 4
CD8	cluster of differentiation 8
cGAMP	cyclic guanosine monophosphate-adenosine monophosphate
cGAS	cGAMP synthase
CpG	cytosine-phosphate-guanine
CH	central helix
COVID-19	Coronavirus Disease 2019
DAMPs	damage-associated molecular patterns
DMV	double-membrane vesicles
DNA	deoxyribonucleic acid
dsRNA	double-stranded RNA
E	envelope
EF	extrafollicular
ER	endoplasmic reticulum
ERGIC	endoplasmic-reticulum-Golgi intermediate compartment
FP	fusion protein
GC	germinal center
HR1	heptad repeat 1
HR2	heptad repeat 2
IFN	interferon
IFNAR	interferon alpha/beta receptor
IFNLR	interferon lambda receptor
IL	interleukin
IP-10	interferon-γ-inducible protein 10
IRF	interferon regulatory factor
ISG	interferon-stimulated gene
ISRE	interferon-stimulated response element
JAK	Janus kinase
M	membrane
MAPK	mitogen-activated protein kinase
MAVS	mitochondrial antiviral-signaling protein
MDA-5	melanoma differentiation-associated protein 5
MHC	major histocompatibility complex
mtDNA	mitochondrial DNA
MyD88	myeloid differentiation primary response 88
N	nucleocapsid
nAbs	Neutralizing antibodies
NLRP3	nucleotide-binding and oligomerization domain-like receptor family pyrin domain containing 3
NF-κB	nuclear factor kappa-light-chain-enhancer of activated B cells
Nsps	nonstructural proteins
NTD	N-terminal domain
Nup98	nucleoporin 98
ORF	open reading frame
PAMPs	pathogen-associated molecular patterns
PLPro	papain-like protease
PRR	pattern recognition receptor
Rae1	ribonucleic acid export 1
RBD	receptor-binding domain
RIG-I	retinoic acid-inducible gene I
RNA	ribonucleic acid
RNase L	ribonuclease L
RTC	replication transcription complex
S	spike
SARS-CoV-2	severe acute respiratory syndrome coronavirus-2
SRP	signal recognition particle
ssRNA	single-stranded RNA
STAT	signal transducer and activator of transcription proteins
STING	stimulator of interferon genes
T_CM_	central memory T cells
T_EM_	effector memory T cells
T_EMRA_	terminally differentiated effector memory T cells
T_FH_	T follicular helper cells
TLRs	toll like receptors
TM	transmembrane domain
TMPRSS2	Transmembrane Serine Protease 2
TNF	tumor necrosis factor
Tom70	translocase of outer membrane 70
TRAF	TNF receptor-associated factor
TRIF	toll/IL-1 receptor protein domain-containing adapter-inducing interferon-β
TRIM	tripartite motif-containing protein 25
T_RM_	Resident memory T cells
UTR	untranslated region
vRNA	viral RNA.
